# Clinical Value of Bronchiectasis and Airspace Enlargement Depicted on Iodine Map in Patients With Interstitial Lung Disease: A Pilot Study

**DOI:** 10.7759/cureus.102934

**Published:** 2026-02-03

**Authors:** Koichiro Yasaka, Sousuke Hatano, Saori Koshino, Go Shirota, Takeyuki Watadani, Osamu Abe

**Affiliations:** 1 Radiology, The University of Tokyo Hospital, Tokyo, JPN; 2 Radiology, National Center for Global Health and Medicine, Tokyo, JPN; 3 Radiology, Graduate School of Medicine, The University of Tokyo, Tokyo, JPN

**Keywords:** dual-energy, dual-energy ct pulmonary angiography, interstitial lung disease (ild), iodine map, multidetector computed tomography, traction bronchiectasis

## Abstract

Objectives

To investigate the degree and clinical meaning of bronchiectasis and airspace enlargement depicted on the iodine map in dual-energy CT pulmonary angiography of patients with interstitial lung disease, as a pilot study.

Methods

This retrospective study included 25 patients with interstitial lung disease who underwent dual-energy CT pulmonary angiography from November 2019 to February 2023. The iodine map and the 70 keV monochromatic image were reconstructed. Three readers independently evaluated the degree of bronchiectasis and airspace enlargement in the iodine map and monochromatic image. The time course of bronchiectasis and airspace enlargement was evaluated by comparing monochromatic images and follow-up conventional CT images for patients with follow-up CT examinations (n = 20).

Results

Bronchiectasis and airspace enlargement in the iodine map were significantly more prominent compared to monochromatic images for all readers (p ≤ 0.030). Patients with histopathologically proven usual interstitial pneumonia and those with rheumatoid arthritis showed image findings progressed in regions where bronchiectasis and airspace enlargement were rated as more prominent compared to monochromatic images by most readers. On the contrary, image finding was ameliorated in those regions in patients with dermatomyositis.

Conclusions

Bronchiectasis and airspace enlargement in the iodine map are depicted more prominently compared to the monochromatic image in dual-energy CT pulmonary angiography and have various time courses based on interstitial lung disease subtypes.

## Introduction

Inflammation or fibrosis within the interstitial space of the lung characterizes interstitial lung disease [1]. Interstitial lung diseases are categorized into several types, including idiopathic, autoimmune-related, and exposure-related diseases. Among idiopathic interstitial lung diseases, usual interstitial pneumonia/idiopathic pulmonary fibrosis is the most prominent and clinically important disease to diagnose, and guidelines regarding idiopathic pulmonary fibrosis have been updated recently [2]. Conversely, the concept of interstitial lung disease has been changing since the emergence of antifibrotic therapy. Antifibrotic therapy is known to slow down lung function decline in patients with idiopathic pulmonary fibrosis [3,4]. Additionally, antifibrotic therapy is effective in patients manifesting progressive pulmonary fibrosis other than idiopathic pulmonary fibrosis [5]. Evaluation of fibrotic features, such as bronchiectasis, reticular abnormality, and honeycombing [2] on CT, plays a crucial role in determining the clinical indication and treatment response for patients with idiopathic pulmonary fibrosis and those with progressive pulmonary fibrosis.

CT technology has been evolving since its introduction, and dual-energy CT is one such relatively new technique. Increasing numbers of research articles revealed the usefulness of dual-energy CT in lung imaging [6-10]. A well-known and successful application is that iodine maps obtainable from dual-energy CT pulmonary angiography depict decreased pulmonary parenchymal perfusion in regions affected by pulmonary embolism. Weidman et al. revealed additional pulmonary embolisms after the iodine map review [9]. The iodine map is also known to be useful in distinguishing acute interstitial pneumonia from lung edema. Takeuchi et al. reported higher mean iodine concentrations in affected areas than in normal areas in patients with acute interstitial lung disease [6]. Researchers have focused on the decrease or increase of quantitatively evaluated iodine concentration in the iodine map, as described above. However, the clinical meaning of morphological information obtainable from the iodine map through qualitative evaluations remains uninvestigated. We serendipitously discovered that bronchiectasis and airspace enlargement are more prominently depicted in the iodine map compared with the monochromatic image in patients with interstitial lung disease. The accurate evaluation of the degree of fibrotic change in patients with interstitial lung disease is essential, especially in the era of antifibrotic therapy for interstitial lung disease; therefore, data regarding the degree of bronchiectasis and airspace enlargement in iodine map compared to the monochromatic image are required, and the clinical value of this imaging feature also needs investigation.

This study aimed to investigate whether bronchiectasis and airspace enlargement depicted in the iodine map are more prominent compared to the monochromatic image in dual-energy CT pulmonary angiography and to evaluate the time course of this feature in follow-up CT examination.

## Materials and methods

The Research Ethics Committee of the Faculty of Medicine of the University of Tokyo issued approval (2561-(29)) and waived the requirement for obtaining written informed consent from patients due to the retrospective study design.

Patients

We searched for all consecutive patients who met the following inclusion criteria: (a) those who underwent dual-energy CT pulmonary angiography for detecting pulmonary embolism with available iodine map and monochromatic images, and (b) those who were clinically or histopathologically diagnosed with interstitial lung disease. Thus, the clinical indication for dual-energy CT pulmonary angiography was the evaluation of pulmonary embolism for all patients. A total of 25 patients met the inclusion criteria from November 2019 to February 2023. Among them, 20 patients had follow-up CT examinations available.

CT examination

All patients underwent dual-energy CT pulmonary angiography with a single CT scanner (Revolution CT, GE Healthcare, Waukesha, WI). Contrast material (600 mgI/kg) was injected from the antecubital vein within 30 seconds using an automatic power injector. This study used the following iodine contrast agents: Iomeron (Bracco, Milan, Italy), Iopamiron (Bayer, Leverkusen, Germany), Omnipaque (GE Healthcare, Waukesha, WI), and Optiray (Guerbet, Villepinte, France). The iodine concentration of the contrast agent was determined based on the body weight as 320, 350, and 370 mgI/ml for <48, 49-63, and >64 kg, respectively. Patients were scanned 20 seconds after initiating the contrast injection. CT scan parameters include the following: tube voltage of fast kVp switching with 80 kVp and 140 kVp; tube current of auto mA with noise index of 11.4; gantry rotation time of 0.5 seconds; pitch of 0.992. The iodine map and 70-keV monochromatic image were reconstructed from the source data. CT attenuation of the monochromatic image of 70 keV is known to correspond to that of a 120-kVp CT image [11]. The slice thickness for both the iodine map and 70-keV monochromatic image was 2.5 mm. The CT scanning and reconstruction parameters for follow-up CT examinations, which are described later, were as follows: tube voltage, 120-140 kVp; tube current, auto mA was used; gantry rotation time, 0.25-0.5 seconds; pitch, 0.813-1.375; and slice thickness, 1-3 mm.

Image analysis

**Figure 1:**
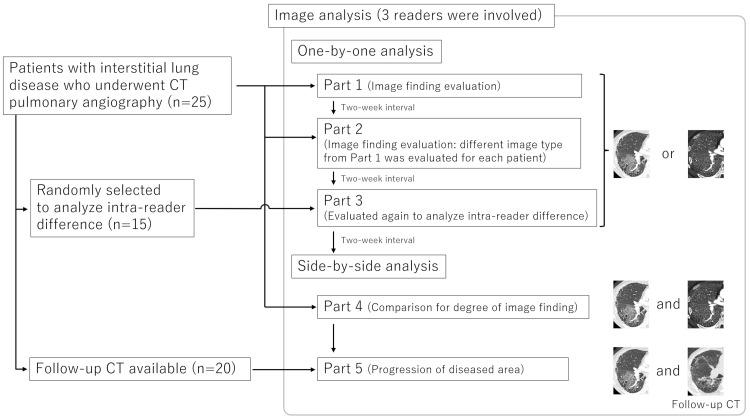
Flowchart of the research protocol.

Three radiologists, with imaging experiences of 13, seven, and three years, performed the qualitative image analysis (Figure 1).

Another radiologist (radiologist A), with an imaging experience of 14 years, randomized all the image sets before the evaluation. Image J (https://imagej.nih.gov/ij/index.html) was used for the image analysis. The readers were blinded to the background information of the patients.

Parts 1 and 2: One-by-One Analysis

Mixed types of image sets (i.e., both iodine map and monochromatic image) were included in each part, while only one image set was included for a single patient. The interval between the evaluations of parts 1 and 2 was two weeks. The readers independently evaluated the images based on the following evaluation items: (a) (for iodine map and monochromatic image) the degree of bronchiectasis and airspace enlargement was compared with background lung parenchyma with a five-point scale (3 = strongly enlarged, 2 = moderately enlarged, 1 = slightly enlarged, 0 = same degree, and −1 = strictured). Radiologist A preselected the region for the evaluation, where bronchiectasis and airspace enlargement were most prominently observed, and was annotated; (b) (for monochromatic image) the presence of honeycomb lung with a five-point scale (5 = present, 4 = probably present, 3 = not sure, 2 = probably not present, and 1 = not present); (c) (for monochromatic image) CT pattern based on the American Thoracic Society (ATS)/European Respiratory Society (ERS)/Japanese Respiratory Society (JRS)/Asociación Latinoamericana de Tórax (ALAT) clinical practice guideline [2] (usual interstitial pneumonia, probable usual interstitial pneumonia, indeterminate for usual interstitial pneumonia, and alternative diagnosis).

Part 3: Intra-reader Difference

In this part, we randomly selected 15 patients from 25 patients to estimate the intra-reader difference and re-evaluated their image sets for bronchiectasis and airspace enlargement by the readers, similarly as in parts 1 and 2. The interval between parts 2 and 3 was two weeks. Selecting part of the subjects in assessing intra-reader agreement is commonly performed in previous studies [12-14].

Part 4: Side-by-Side Analysis (Iodine Map vs. Monochromatic Image)

While the one-by-one analysis enabled bias reduction in comparing bronchiectasis between the iodine map and the monochromatic image, both the iodine map and the monochromatic image are referenced in making the diagnosis in actual clinical practice. Therefore, the readers were also involved in the side-by-side qualitative image analysis, two weeks after part 3. They evaluated the images in terms of the following: degree of bronchiectasis and airspace enlargement in the preselected region as in parts 1 and 2 by comparing iodine map and monochromatic images with a five-point scale (2 = iodine map is strongly prominent, 1 = iodine map is prominent, 0 = same degree, −1 = monochromatic image is prominent, and −2 = monochromatic image is strongly prominent).

Part 5: Side-by-Side Analysis (Monochromatic Image vs. Follow-Up CT Image)

In this part, the readers evaluated the images in terms of the following by comparing monochromatic images and follow-up single-energy CT images: progression of image finding in the preselected region (2 = ameliorated, 1 = slightly ameliorated, 0 = no change, −1 = slightly progressed, and −2 = progressed).

Statistical analysis

R version 4.1.2 (https://www.r-project.org/; R Foundation for Statistical Computing, Vienna, Austria) was used for statistical analyses. One-by-one, bronchiectasis and airspace enlargement scores were compared between the iodine map and monochromatic image with Wilcoxon’s signed rank test. Intra-reader and inter-reader agreement are crucial issues in evaluating interstitial lung disease image findings [15]. We calculated the difference in scores instead of calculating Cohen’s weighted kappa values for the intra-reader and inter-reader agreement of bronchiectasis and airspace enlargement [16]. This was because the range of bronchiectasis and airspace enlargement scores in the iodine map (1-3) was narrower than that in monochromatic images (0-3), in which case the κ value is known to show a paradoxically low value [17]. The mean scores for all readers of <0, 0, and >0 were considered to indicate progress, no change, and amelioration, respectively, in judging the image finding progression.

## Results

Patients

This study included 25 patients, including 11 males (mean age: 68.8 ± 11.1 years) and 14 females (mean age: 69.9 ± 11.4 years). The median follow-up interval (with interquartile range) for those with available follow-up CT examination data was 164 (97.8-392.3) days. Among 25 patients, two patients with histopathologically diagnosed usual interstitial pneumonia were treated with antifibrotic therapy.

Evaluations of bronchiectasis and airspace enlargement in iodine map and monochromatic images

Table 1 shows the detailed results for one-by-one bronchiectasis and airspace enlargement scores. All readers rated that bronchiectasis and airspace enlargement are significantly more prominent in the iodine map compared to monochromatic images (p < 0.030). Table 2 shows the intra-reader and inter-reader differences in scores. The difference in scores was ≤2 for all patients. Intra-reader and inter-reader differences demonstrated no statistical significance in the scores between iodine map and monochromatic images (p ≥ 0.073 and p ≥ 0.084, respectively).

**Table 1:** Readers’ scores for the degree of bronchiectasis and airspace enlargement in one-by-one analysis (parts 1 and 2). The number of patients for score 3 (strongly enlarged)/2 (moderately enlarged)/1 (slightly enlarged)/0 (same degree)/−1 (strictured) is shown. Wilcoxon’s signed rank test was performed for comparison. * Statistically significant difference (p < 0.050). All readers rated that bronchiectasis and airspace enlargement were more strongly observed in the iodine map compared to the monochromatic image.

Image	Reader 1	Reader 2	Reader 3
Monochromatic image	7/7/6/5/0	11/5/3/6/0	6/7/4/8/0
Iodine map	13/9/3/0/0	14/8/3/0/0	8/13/4/0/0
Comparison	0.006*	0.030*	0.004*

**Table 2:** Intra-reader (part 3) and inter-reader (part 1 and 2) differences in scores for bronchiectasis and airspace enlargement. The number of patients for absolute score differences 4/3/2/1/0 is shown. A larger absolute score difference indicates greater intra-reader difference or inter-reader difference. Wilcoxon’s signed rank test was performed for comparison. There was no statistically significant difference in intra-reader and inter-reader differences in the evaluations of bronchiectasis and airspace enlargement between the monochromatic image and the iodine map.

	Intra-reader			Inter-reader		
Image	Reader 1	Reader 2	Reader 3	Readers 1 vs. 2	Readers 2 vs. 3	Readers 3 vs. 1
Monochromatic image	0/0/1/6/8	0/0/0/6/9	0/0/1/9/5	0/0/1/9/15	0/0/4/10/11	0/0/3/9/13
Iodine map	0/0/0/5/10	0/0/0/11/4	0/0/0/6/9	0/0/3/13/9	0/0/1/9/15	0/0/1/14/10
Comparison	0.149	0.073	0.183	0.084	0.178	0.857

Subgroup analysis: Pathologically proven usual interstitial pneumonia

**Figure 2:**
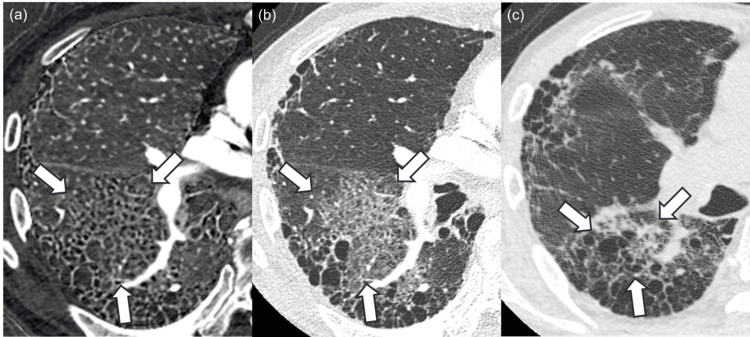
Images of a patient histopathologically diagnosed with usual interstitial pneumonia. The iodine map (a) and monochromatic image (b) of a 77-year-old male patient histopathologically diagnosed with usual interstitial pneumonia. Bronchiectasis and airspace enlargement, which are presented as black tiny structures in the region surrounded by white arrows, are more prominent in the iodine map compared to the monochromatic image, and readers 1, 2, and 3 rated 1 (iodine map is prominent), 0 (same degree), and 2 (iodine map is strongly prominent) in side-by-side qualitative evaluation, respectively. The region surrounded by the white arrows contracted in the follow-up CT examination (c), which was performed after 25 days. Window level/width for iodine map (a) and CT images (b, c) were 15/110 mgI/ml and –600/1600 Hounsfield unit, respectively.

Among 25 patients, four were histopathologically diagnosed with usual interstitial pneumonia (Figure 2).

In these patients, the numbers of those pooled for the three readers in side-by-side bronchiectasis and airspace enlargement score (2 (iodine map >> monochromatic image)/1 (iodine map > monochromatic image)/0 (same degree)/−1 (iodine map < monochromatic image)/−2 (iodine map << monochromatic image)), one-by-one honeycomb lung score (5 (present)/4 (probably present)/3 (not sure)/2 (probably not present)/1 (not present)), and usual interstitial pneumonia diagnosis (usual interstitial pneumonia/probably/indeterminate/alternative) were 5/4/3/0/0, 7/2/2/0/1, and 7/0/4/1, respectively (Table 3).

**Table 3:** The results of image analysis. The number of patients for each score, which is pooled for three readers, is shown in each cell.

	Pathologically proven usual interstitial pneumonia	Rheumatoid arthritis	Systemic sclerosis	Dermatomyositis	Others
Side-by-side bronchiectasis and airspace enlargement score					
2 (iodine map >> monochromatic image)	5	2	8	5	12
1 (iodine map > monochromatic image)	4	3	7	3	15
0 (same degree)	3	1	1	1	1
-1 (iodine map < monochromatic image)	0	0	1	0	1
-2 (iodine map << monochromatic image)	0	0	1	0	1
Honeycomb lung score on monochromatic image					
5 (present)	7	0	6	1	5
4 (probably present)	2	0	2	1	0
3 (not sure)	2	1	1	2	2
2 (probably not present)	0	2	1	0	0
1 (not present)	1	3	8	5	23
CT pattern on monochromatic image					
Usual interstitial pneumonia	7	0	7	0	4
Probable usual interstitial pneumonia	0	0	2	1	2
Indeterminate for usual interstitial pneumonia	4	2	1	5	4
Alternative diagnosis	1	4	8	3	20

Follow-up CT was available, and image finding progressed in the follow-up CT examination for all of these patients.

Subgroup analysis: Rheumatoid arthritis

**Figure 3:**
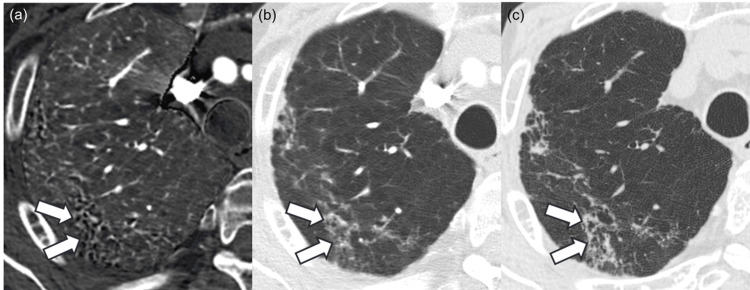
Images of a patient diagnosed with rheumatoid arthritis. The iodine map (a) and monochromatic image (b) of a 79-year-old male patient diagnosed with rheumatoid arthritis. Bronchiectasis and airspace enlargement, which are presented as black tiny structures in the region surrounded by white arrows, are more prominent in the iodine map compared to the monochromatic image, and readers 1, 2, and 3 rated 2 (iodine map is strongly prominent), 1 (iodine map is prominent), and 2 in side-by-side qualitative evaluation, respectively. Bronchiectasis and airspace enlargement progressed in the evaluated region in the follow-up CT examination (c), which was performed after 127 days. Window level/width for iodine map (a) and CT images (b, c) were 15/110 mgI/ml and –600/1600 Hounsfield unit, respectively.

Two patients were diagnosed with rheumatoid arthritis (Figure 3).

In these patients, the numbers of those pooled for the three readers in side-by-side bronchiectasis and airspace enlargement score (2 (iodine map >> monochromatic image)/1 (iodine map > monochromatic image)/0 (same degree)/−1 (iodine map < monochromatic image)/−2 (iodine map << monochromatic image)), one-by-one honeycomb lung score (5 (present)/4 (probably present)/3 (not sure)/2 (probably not present)/1 (not present)), and usual interstitial pneumonia diagnosis (usual interstitial pneumonia/probably/indeterminate/alternative) were 2/3/1/0/0, 0/0/1/2/3, and 0/0/2/4, respectively (Table 3). Follow-up CT was available, and image finding progressed in the follow-up CT examination for both patients.

Subgroup analysis: Systemic sclerosis

**Figure 4:**
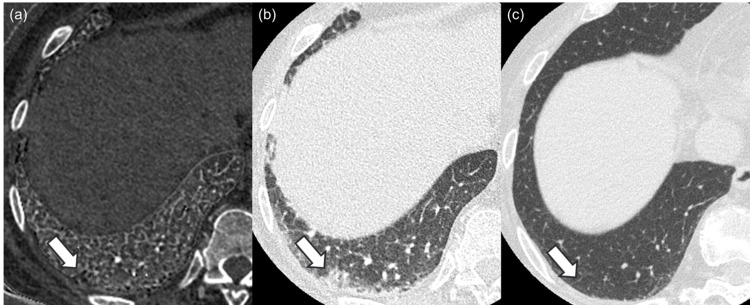
Images of a patient diagnosed with systemic sclerosis. The iodine map (a) and monochromatic image (b) of a 61-year-old female patient diagnosed with systemic sclerosis. Bronchiectasis and airspace enlargement, which are presented as black tiny structures in the region surrounded by white arrows, are more prominent in the iodine map compared to the monochromatic image, and readers 1, 2, and 3 rated 1 (iodine map is prominent), 1, and 2 (iodine map is strongly prominent) in side-by-side qualitative evaluation, respectively. Bronchiectasis and airspace enlargement were ameliorated in the evaluated region in the follow-up CT examination (c), which was performed after 490 days. Window level/width for iodine map (a) and CT images (b, c) were 15/110 mgI/ml and –600/1600 Hounsfield unit, respectively.

Six patients were diagnosed with systemic sclerosis (Figure 4).

In these patients, the numbers of those pooled for the three readers in side-by-side bronchiectasis and airspace enlargement score (2 (iodine map >> monochromatic image)/1 (iodine map > monochromatic image)/0 (same degree)/−1 (iodine map < monochromatic image)/−2 (iodine map << monochromatic image)), one-by-one honeycomb lung score (5 (present)/4 (probably present)/3 (not sure)/2 (probably not present)/1 (not present)), and usual interstitial pneumonia diagnosis (usual interstitial pneumonia/probably/indeterminate/alternative) were 8/7/1/1/1, 6/2/1/1/8, and 7/2/1/8, respectively (Table 3). Follow-up CT was available for four patients, and image finding was progressed and ameliorated in the follow-up CT examination for two and two patients, respectively.

Subgroup analysis: Dermatomyositis

**Figure 5:**
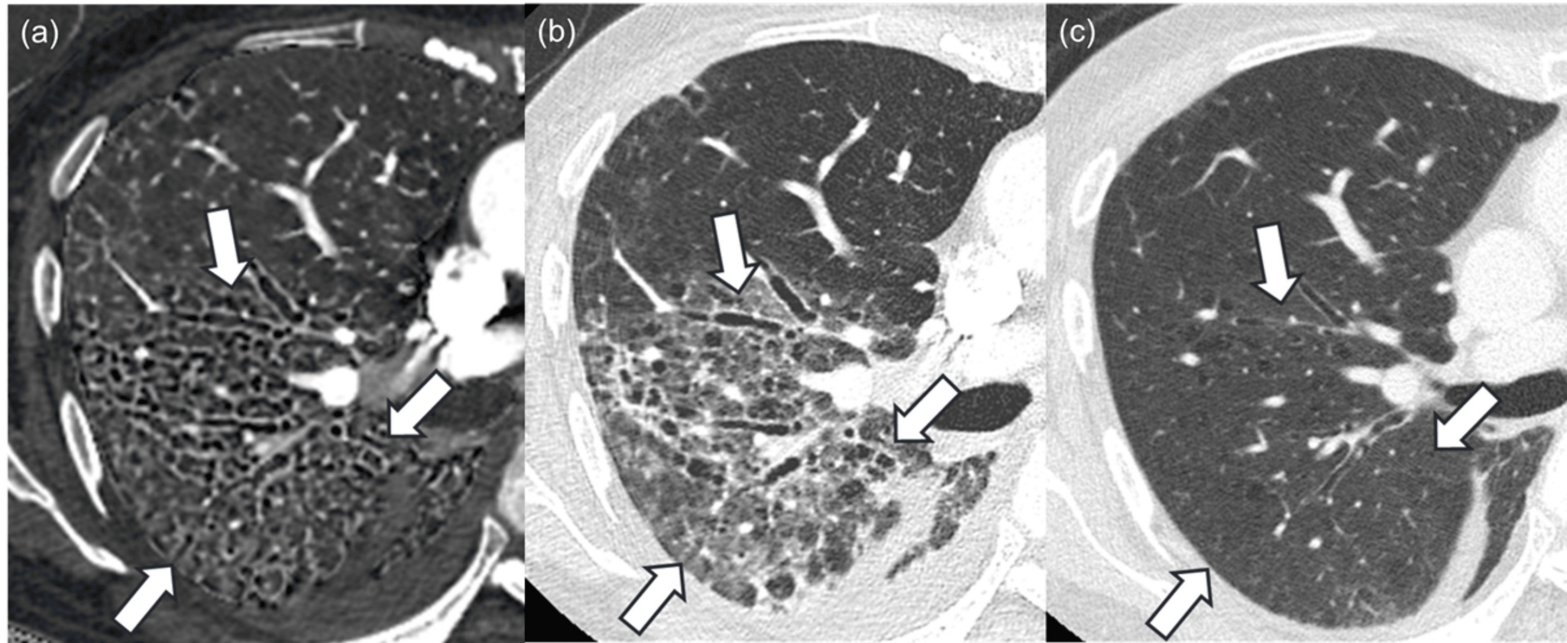
Images of a patient diagnosed with antisynthetase syndrome. The iodine map (a) and monochromatic image (b) of a 72-year-old female patient diagnosed with antisynthetase syndrome. Bronchiectasis and airspace enlargement, which are presented as black tiny structures in the region surrounded by white arrows, are more prominent in the iodine map compared to the monochromatic image, and readers 1, 2, and 3 rated 0 (same degree), 1 (iodine map is prominent), and 1 in side-by-side qualitative evaluation, respectively. Bronchiectasis and airspace enlargement were ameliorated in the evaluated region in the follow-up CT examination (c), which was performed after 100 days. Window level/width for iodine map (a) and CT images (b, c) were 15/110 mgI/ml and –600/1600 Hounsfield unit, respectively.

**Figure 6:**
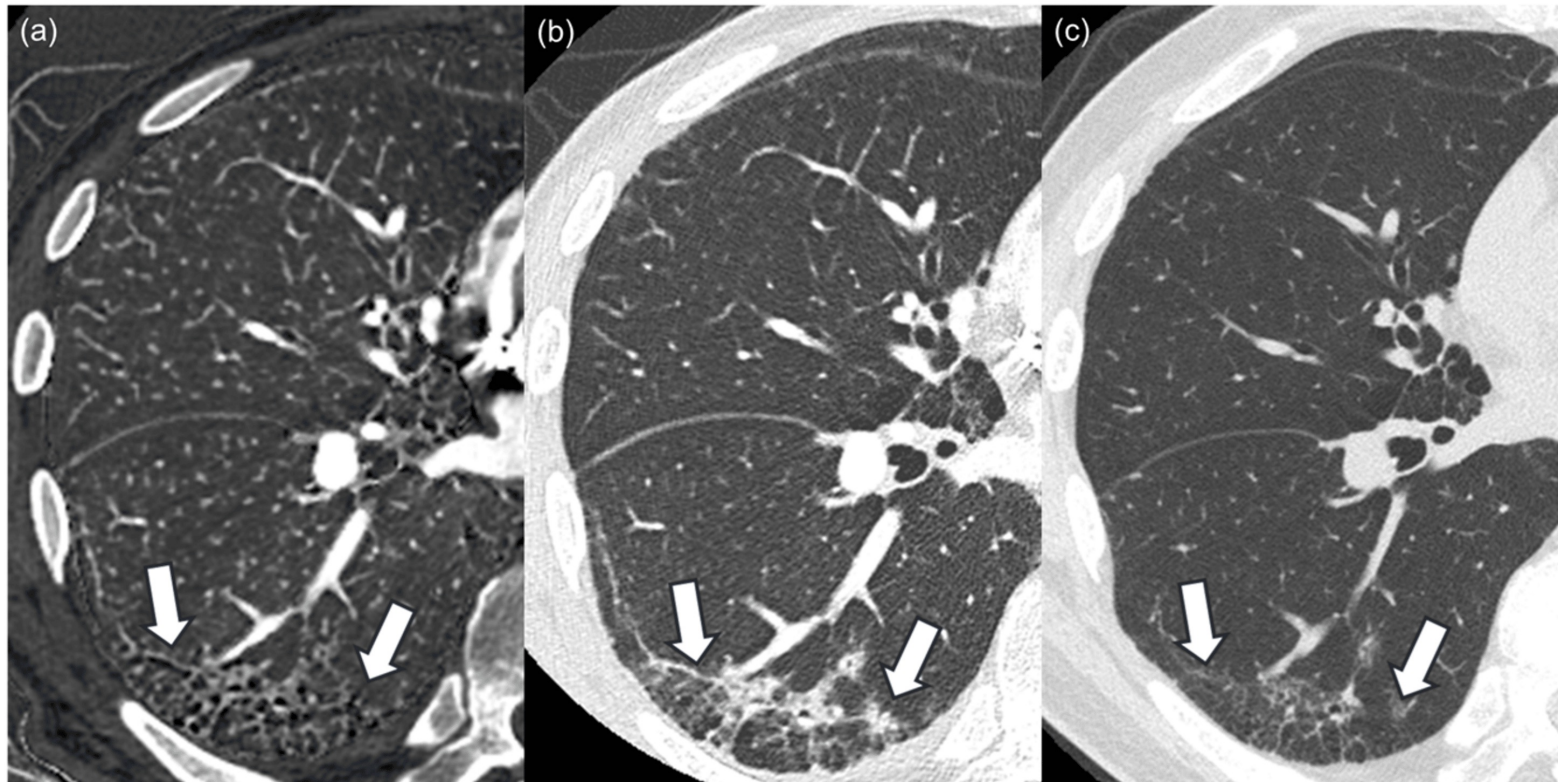
Images of a patient diagnosed with anti-melanoma differentiation-associated gene 5 antibody-positive dermatomyositis. The iodine map (a) and monochromatic image (b) of a 55-year-old female patient diagnosed with anti-melanoma differentiation-associated gene 5 antibody-positive dermatomyositis. Bronchiectasis and airspace enlargement, which are presented as black tiny structures in the region surrounded by white arrows, are more prominent in the iodine map compared to the monochromatic image, and readers 1, 2, and 3 rated 2 (iodine map is strongly prominent) in side-by-side qualitative evaluation. Bronchiectasis and airspace enlargement were ameliorated in the evaluated region in the follow-up CT examination (c), which was performed after 468 days. Window level/width for iodine map (a) and CT images (b, c) were 15/110 mgI/ml and –600/1600 Hounsfield unit, respectively.

Three patients were diagnosed with dermatomyositis. Among them, one patient was positive for anti-aminoacyl-tRNA synthetase antibody (Figure 5), and one patient was positive for melanoma differentiation-associated gene 5 antibodies (Figure 6).

The numbers of patients pooled for the three readers in side-by-side bronchiectasis and airspace enlargement score (2 (iodine map >> monochromatic image)/1 (iodine map > monochromatic image)/0 (same degree)/−1 (iodine map < monochromatic image)/−2 (iodine map << monochromatic image)), one-by-one honeycomb lung score (5 (present)/4 (probably present)/3 (not sure)/2 (probably not present)/1 (not present)), and usual interstitial pneumonia diagnosis (usual interstitial pneumonia/probably/indeterminate/alternative) were 5/3/1/0/0, 1/1/2/0/5, and 0/1/5/3, respectively (Table 3). Follow-up CT was available, and image finding was ameliorated in the follow-up CT examination for all of these patients.

Subgroup analysis: Others

In the other 10 patients, one was histopathologically diagnosed with pleuroparenchymal fibroelastosis, one with systemic lupus erythematosus, and one with immunoglobulin G4-associated disease. The numbers of patients pooled for the three readers in side-by-side bronchiectasis and airspace enlargement score (2 (iodine map >> monochromatic image)/1 (iodine map > monochromatic image)/0 (same degree)/−1 (iodine map < monochromatic image)/−2 (iodine map << monochromatic image)), one-by-one honeycomb lung score (5 (present)/4 (probably present)/3 (not sure)/2 (probably not present)/1 (not present)), and usual interstitial pneumonia diagnosis (usual interstitial pneumonia/probably/indeterminate/alternative) were 12/15/1/1/1, 5/0/2/0/23, and 4/2/4/20, respectively (Table 3). Follow-up CT was available for seven patients, and image finding was progressed, stable, and ameliorated in the follow-up CT examination for four, one, and two patients, respectively.

## Discussion

The usefulness of iodine concentration on the iodine map in evaluating lung diseases is a well-known fact, while the clinical meaning of morphologic features of interstitial lung disease on the iodine map, which can only be qualitatively evaluated, remains uninvestigated. This study revealed that bronchiectasis and airspace enlargement are more prominently seen in the iodine map compared to the monochromatic image on dual-energy CT pulmonary angiography. Additionally, the time course of bronchiectasis and airspace enlargement in follow-up CT examination differed across patients with different interstitial lung disease subtypes.

We included three readers with various imaging experiences in evaluating bronchiectasis and airspace enlargement. All readers rated this feature to be significantly more prominently depicted in the iodine map compared to the monochromatic image. This could be because the contrast agent does not distribute within exudates in the airway. Therefore, an enlarged airway is depicted as dark intensity in the iodine map, while exudates result in ground glass opacities or consolidation in a monochromatic image. Another possible reason may be that inflammation increased the blood flow of the interstitial space, causing higher intensity in the iodine map. Because the iodine map is a calculated image obtained via image processing, technological issues might also explain the image finding within the diseased region.

Bronchiectasis and airspace enlargement in the iodine map were more prominently depicted compared with monochromatic images for most patients with histopathologically proven usual interstitial pneumonia and those with rheumatoid arthritis. Image finding in the evaluated regions progressed in follow-up CT examination in all these patients. On the contrary, image finding was ameliorated in follow-up CT examination for all patients with dermatomyositis, while bronchiectasis and airspace enlargement in the iodine map were more prominent compared with the monochromatic image for most of them. Patients with systemic sclerosis and others demonstrated mixed ratings for bronchiectasis and airspace enlargement, as well as a mixed time course for image finding. These results indicate the importance of knowing the interstitial lung disease subtype and understanding its time course to interpret the meaning of bronchiectasis and airspace enlargement in the iodine map. Antifibrotic therapy slows down lung function decline [3,4], and idiopathic pulmonary fibrosis fundamentally has a progressive course [18]. Acute exacerbations, in which hyaline membrane formation is observed in the airway, can also occur in patients with idiopathic pulmonary fibrosis. Usual interstitial pneumonia or usual interstitial pneumonia-like pattern is found in 70-80% of patients with rheumatoid arthritis-associated interstitial lung disease, whereas other connective tissue diseases show a nonspecific interstitial pneumonia pattern as the predominant CT finding [19]. Patients with usual interstitial pneumonia and rheumatoid arthritis prominently demonstrated bronchiectasis and airspace enlargement in the iodine map, indicating a high risk of image finding deterioration within this region in the follow-up CT. This feature does not necessarily indicate future image finding progression in patients diagnosed with conditions other than usual interstitial pneumonia or rheumatoid arthritis.

This study has some limitations. First, this study included a relatively small number of patients. However, a statistically significant difference could be observed in the degree of bronchiectasis and airspace enlargement between the iodine map and the monochromatic image. Second, histopathological diagnosis data were not necessarily available for all patients. Future studies, ideally including histopathological evaluations, are needed to investigate the precise mechanism underlying our study results. Third, it is not common to perform dual-energy CT pulmonary angiography for evaluations of interstitial lung disease. We think it would be better to evaluate whether bronchiectasis and airspace enlargement are present when patients with interstitial lung disease undergo dual-energy CT pulmonary angiography for other purposes. However, CT requires radiation exposure to patients. Therefore, some other studies regarding similar topics in relation to the adverse reactions of radiation exposure would be needed to make clear whether dual-energy CT pulmonary angiography needs to be added solely to evaluate the presence of bronchiectasis and airspace enlargement on iodine map. Fourth, radiologist A selected the region for evaluation during image analysis, which could lead to bias. Fifth, quantitative evaluations were not performed because measurements using regions of interest on the bronchi may not yield sufficiently reliable results due to their small size. Finally, this study used a single CT scanner. Our study results would not necessarily apply to CT data obtained with other vendor machines because the techniques for obtaining dual-energy CT data are different across CT vendors.

## Conclusions

In conclusion, bronchiectasis and airspace enlargement, which can only be qualitatively evaluated, were more prominently seen in the iodine map compared with the monochromatic image. Morphological information in the iodine map has not been investigated so far. The time course of bronchiectasis and airspace enlargement was variable depending on the interstitial lung disease subtype. While conventional CT images provide information regarding the differential diagnosis of various types of interstitial lung diseases, we need to consider the morphologic features of the lung on the iodine map in estimating the progression of the diseased region. Our study results suggest performing CT pulmonary angiography with dual-energy CT, if available, in patients with interstitial lung disease suspected of pulmonary embolism. Because both radiologists and radiology technologists are involved in assigning CT protocols, our study findings would benefit not only radiologists but also radiology technologists. Lastly, because the present study was a pilot study, further investigation is required to determine whether it influences clinical decision-making or can predict disease progression.
